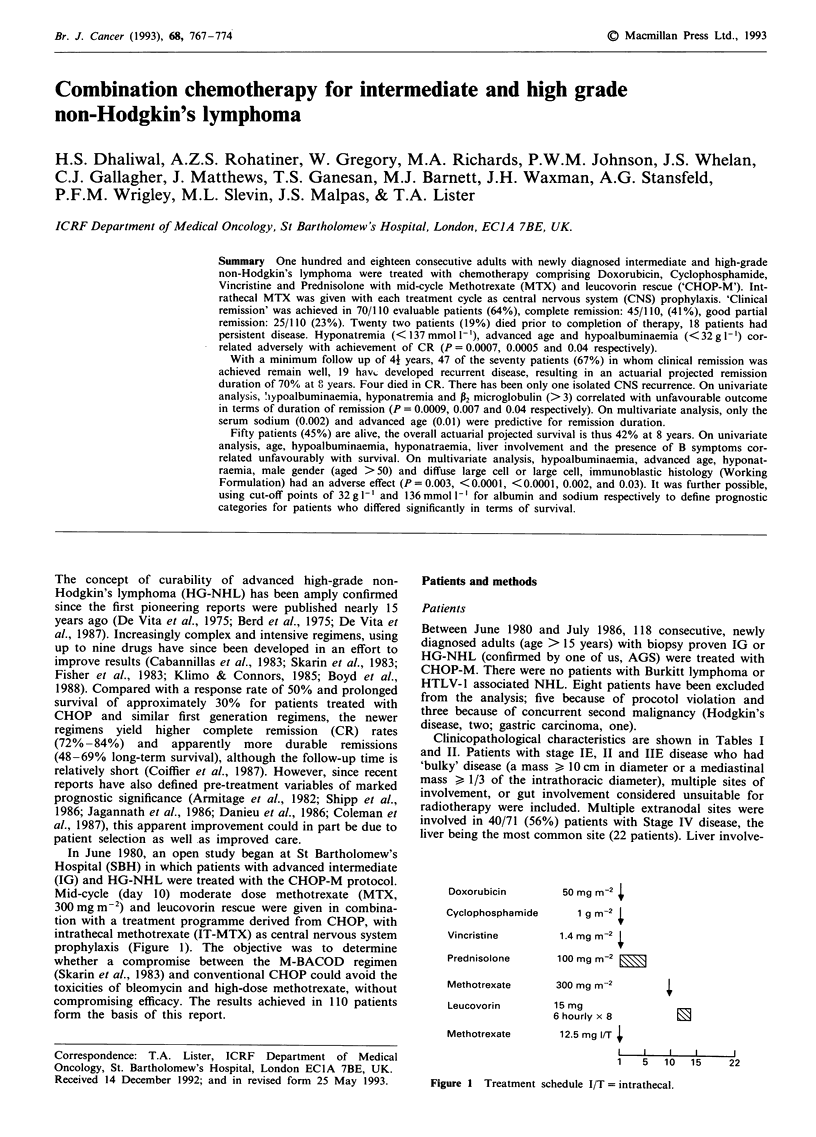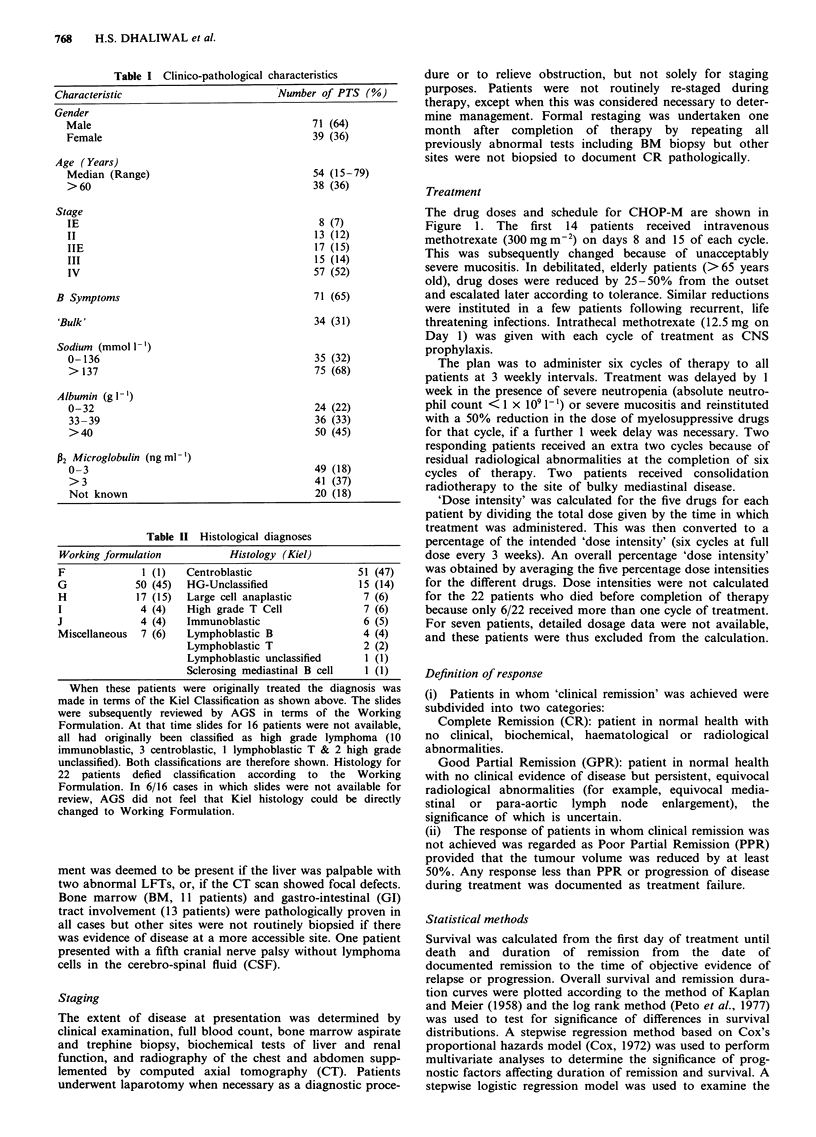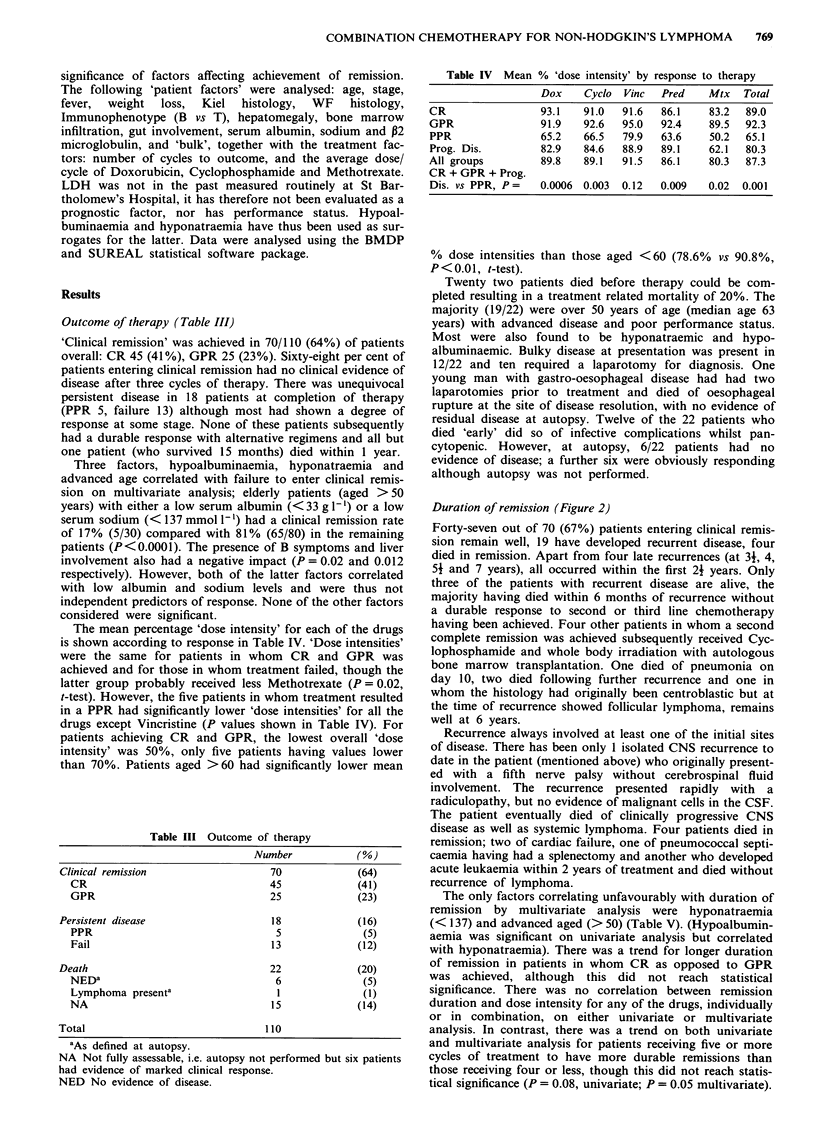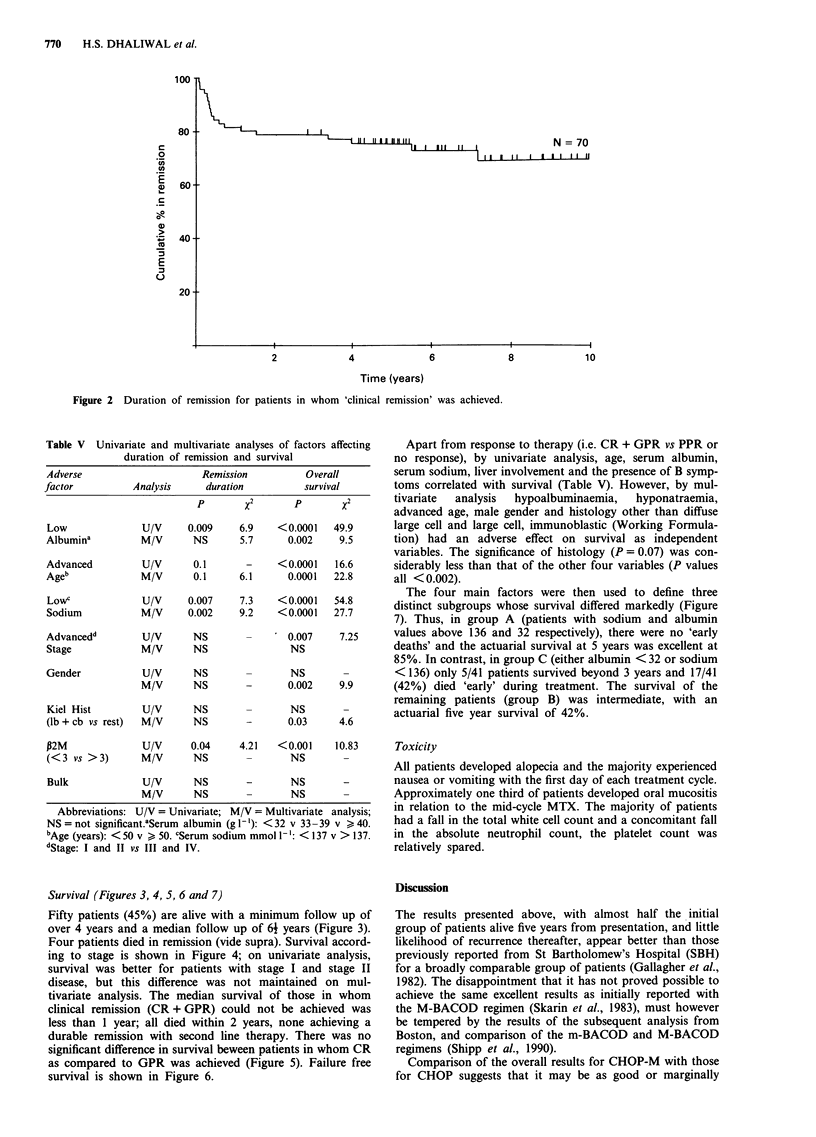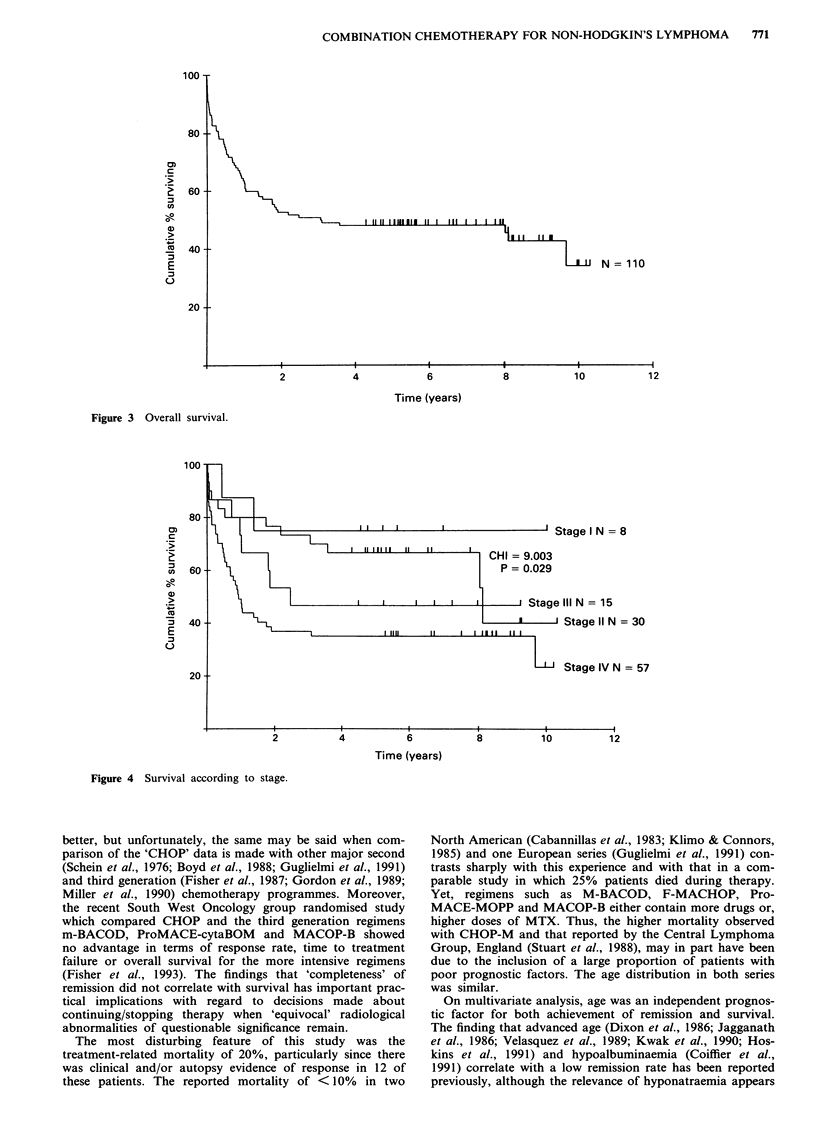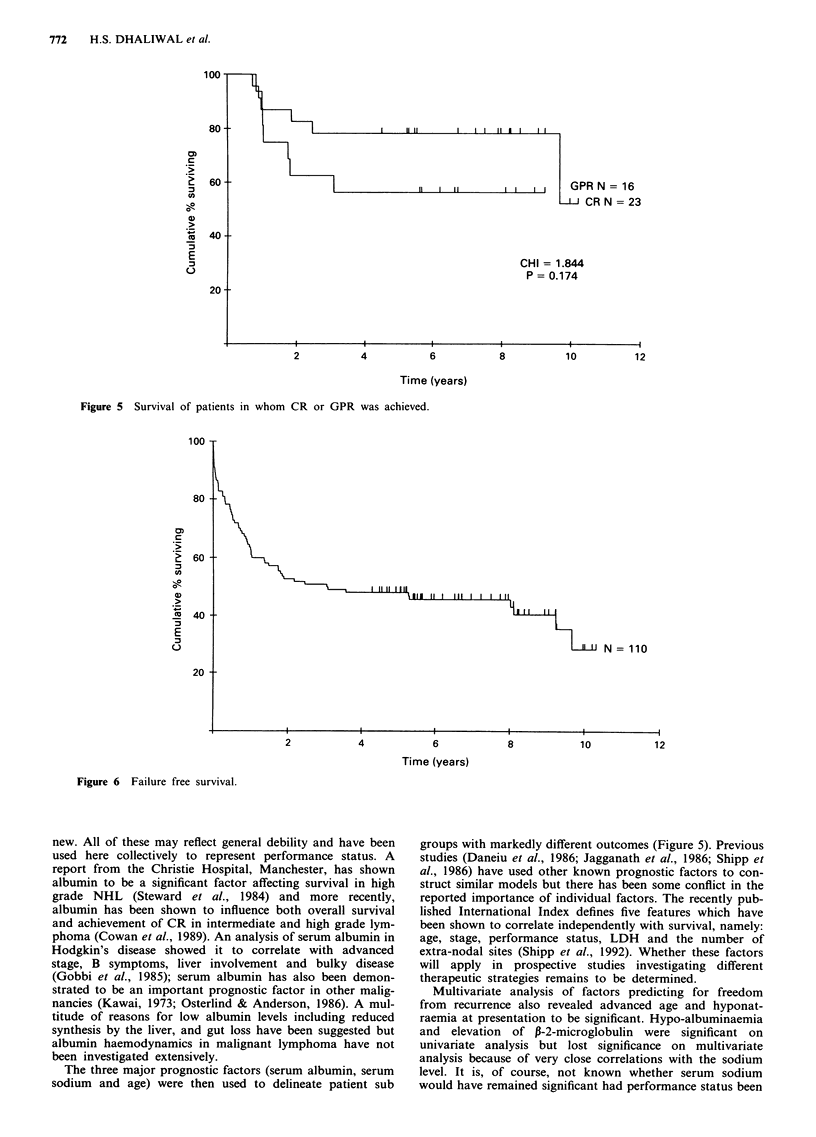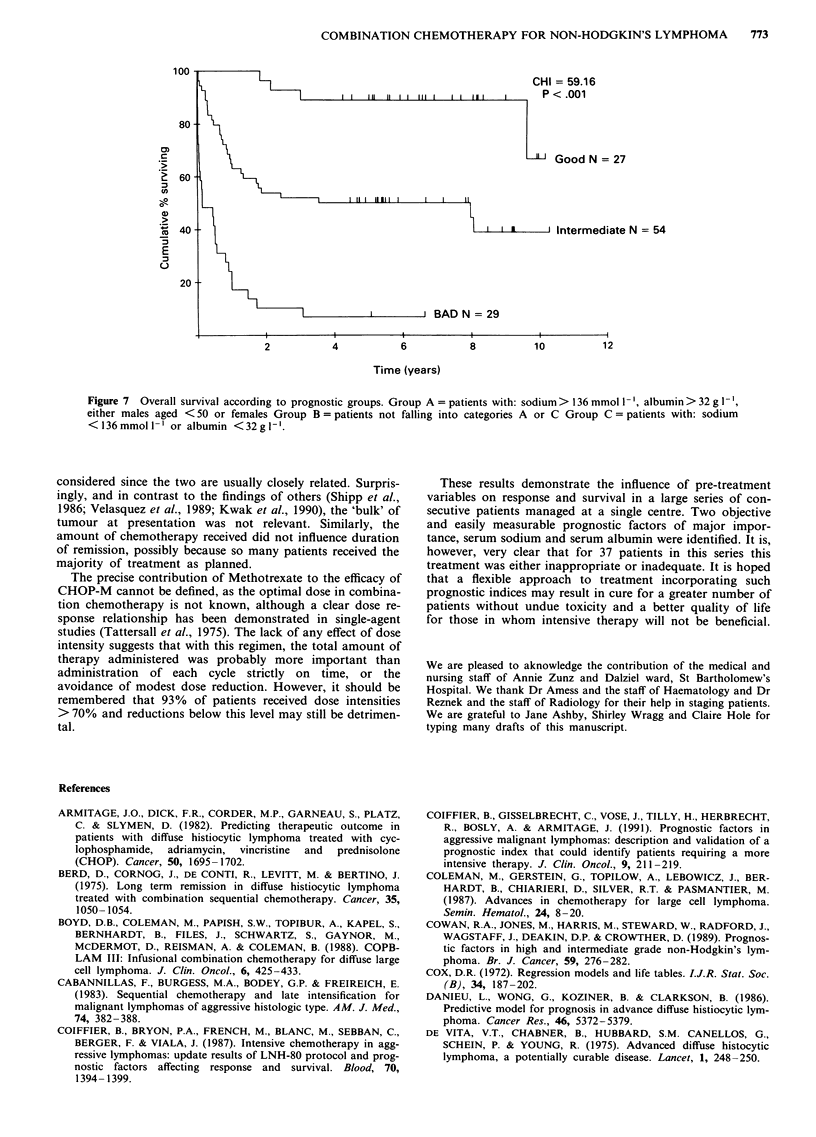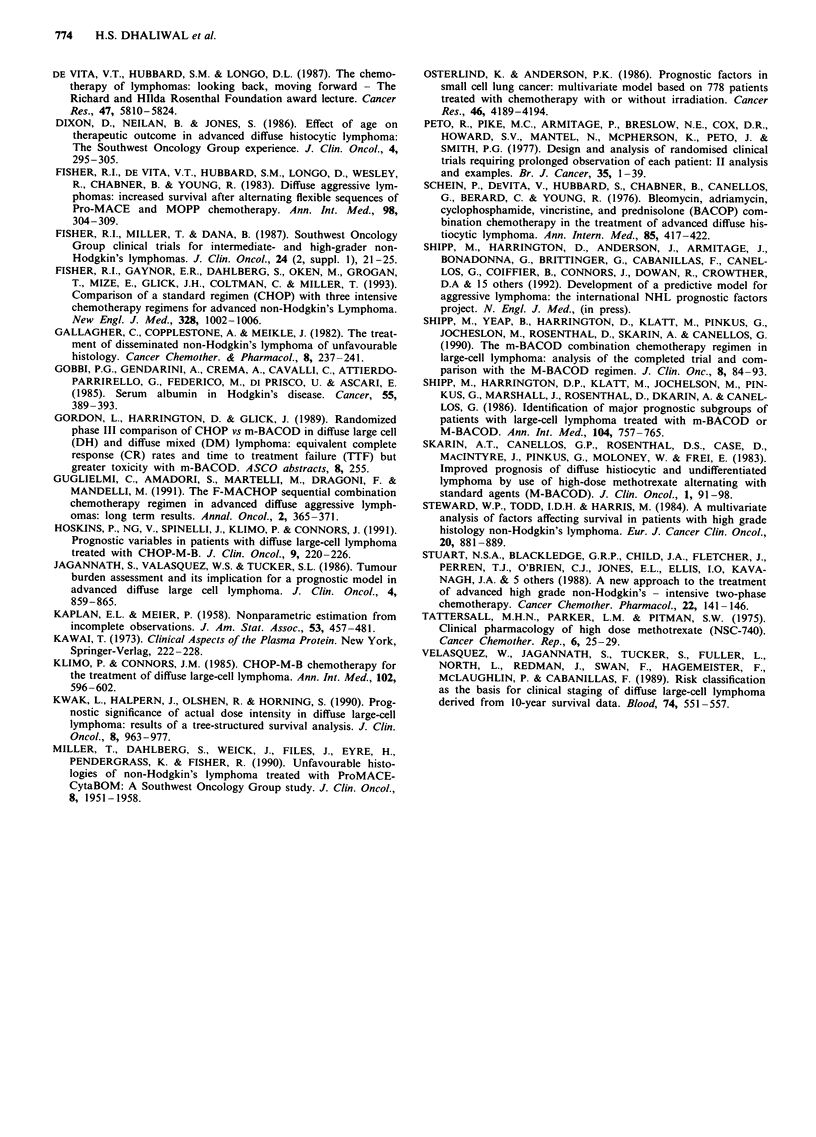# Combination chemotherapy for intermediate and high grade non-Hodgkin's lymphoma.

**DOI:** 10.1038/bjc.1993.425

**Published:** 1993-10

**Authors:** H. S. Dhaliwal, A. Z. Rohatiner, W. Gregory, M. A. Richards, P. W. Johnson, J. S. Whelan, C. J. Gallagher, J. Matthews, T. S. Ganesan, M. J. Barnett

**Affiliations:** ICRF Department of Medical Oncology, St Bartholomew's Hospital, London, UK.

## Abstract

One hundred and eighteen consecutive adults with newly diagnosed intermediate and high-grade non-Hodgkin's lymphoma were treated with chemotherapy comprising Doxorubicin, Cyclophosphamide, Vincristine and Prednisolone with mid-cycle Methotrexate (MTX) and leucovorin rescue ('CHOP-M'). Intrathecal MTX was given with each treatment cycle as central nervous system (CNS) prophylaxis. 'Clinical remission' was achieved in 70/110 evaluable patients (64%), complete remission: 45/110, (41%), good partial remission: 25/110 (23%). Twenty two patients (19%) died prior to completion of therapy, 18 patients had persistent disease. Hyponatremia (< 137 mmol l-1), advanced age and hypoalbuminaemia (< 32 g l-1) correlated adversely with achievement of CR (P = 0.0007, 0.0005 and 0.04 respectively). With a minimum follow up of 41 years, 47 of the seventy patients (67%) in whom clinical remission was achieved remain well, 19 have developed recurrent disease, resulting in an actuarial projected remission duration of 70% at 8 years. Four died in CR. There has been only one isolated CNS recurrence. On univariate analysis, hypoalbuminaemia, hyponatremia and beta 2 microglobulin (> 3) correlated with unfavourable outcome in terms of duration of remission (P = 0.0009, 0.007 and 0.04 respectively). On multivariate analysis, only the serum sodium (0.002) and advanced age (0.01) were predictive for remission duration. Fifty patients (45%) are alive, the overall actuarial projected survival is thus 42% at 8 years. On univariate analysis, age, hypoalbuminaemia, hyponatraemia, liver involvement and the presence of B symptoms correlated unfavourably with survival. On multivariate analysis, hypoalbuminaemia, advanced age, hyponatraemia, male gender (aged > 50) and diffuse large cell or large cell, immunoblastic histology (Working Formulation) had an adverse effect (P = 0.003, < 0.0001, < 0.0001, 0.002, and 0.03). It was further possible, using cut-off points of 32 g l-1 and 136 mmol l-1 for albumin and sodium respectively to define prognostic categories for patients who differed significantly in terms of survival.


*Br. J. Cancer* (1993), **68**, 767–774 © Macmillan Press Ltd., 1993

### Combination chemotherapy for intermediate and high grade non-Hodgkin's lymphoma

H.S. Dhaliwal, A.Z.S. Rohatiner, W. Gregory, M.A. Richards, P.W.M. Johnson, J.S. Whelan, C.J. Gallagher, J. Matthews, T.S. Ganesan, M.J. Barnett, J.H. Waxman, A.G. Stansfeld, P.F.M. Wrigley, M.L. Slevin, J.S. Malpas, & T.A. Lister

*ICRF Department of Medical Oncology, St Bartholomew's Hospital, London, EC1A 7BE, UK.*

**Summary** One hundred and eighteen consecutive adults with newly diagnosed intermediate and high-grade non-Hodgkin's lymphoma were treated with chemotherapy comprising Doxorubicin, Cyclophosphamide, Vincristine and Prednisolone with mid-cycle Methotrexate (MTX) and leucovorin rescue ('CHOP-M'). Intrathecal MTX was given with each treatment cycle as central nervous system (CNS) prophylaxis. 'Clinical remission' was achieved in 70/110 evaluable patients (64%), complete remission: 45/110, (41%), good partial remission: 25/110 (23%). Twenty two patients (19%) died prior to completion of therapy, 18 patients had persistent disease. Hyponatremia ($<137\ mmol\ l^{-1}$), advanced age and hypoalbuminaemia ($<32\ g\ l^{-1}$) correlated adversely with achievement of CR ($P = 0.0007$, 0.0005 and 0.04 respectively).

With a minimum follow up of 4½ years, 47 of the seventy patients (67%) in whom clinical remission was achieved remain well, 19 have developed recurrent disease, resulting in an actuarial projected remission duration of 70% at 8 years. Four died in CR. There has been only one isolated CNS recurrence. On univariate analysis, hypoalbuminaemia, hyponatremia and $\beta_2$ microglobulin ($>3$) correlated with unfavourable outcome in terms of duration of remission ($P = 0.0009$, 0.007 and 0.04 respectively). On multivariate analysis, only the serum sodium (0.002) and advanced age (0.01) were predictive for remission duration.

Fifty patients (45%) are alive, the overall actuarial projected survival is thus 42% at 8 years. On univariate analysis, age, hypoalbuminaemia, hyponatraemia, liver involvement and the presence of B symptoms correlated unfavourably with survival. On multivariate analysis, hypoalbuminaemia, advanced age, hyponatraemia, male gender (aged $>50$) and diffuse large cell or large cell, immunoblastic histology (Working Formulation) had an adverse effect ($P = 0.003$, $<0.0001$, $<0.0001$, 0.002, and 0.03). It was further possible, using cut-off points of $32\ g\ l^{-1}$ and $136\ mmol\ l^{-1}$ for albumin and sodium respectively to define prognostic categories for patients who differed significantly in terms of survival.

The concept of curability of advanced high-grade non-Hodgkin's lymphoma (HG-NHL) has been amply confirmed since the first pioneering reports were published nearly 15 years ago (De Vita *et al.*, 1975; Berd *et al.*, 1975; De Vita *et al.*, 1987). Increasingly complex and intensive regimens, using up to nine drugs have since been developed in an effort to improve results (Cabannillas *et al.*, 1983; Skarin *et al.*, 1983; Fisher *et al.*, 1983; Klimo & Connors, 1985; Boyd *et al.*, 1988). Compared with a response rate of 50% and prolonged survival of approximately 30% for patients treated with CHOP and similar first generation regimens, the newer regimens yield higher complete remission (CR) rates (72%–84%) and apparently more durable remissions (48–69% long-term survival), although the follow-up time is relatively short (Coiffier *et al.*, 1987). However, since recent reports have also defined pre-treatment variables of marked prognostic significance (Armitage *et al.*, 1982; Shipp *et al.*, 1986; Jagannath *et al.*, 1986; Danieu *et al.*, 1986; Coleman *et al.*, 1987), this apparent improvement could in part be due to patient selection as well as improved care.

In June 1980, an open study began at St Bartholomew's Hospital (SBH) in which patients with advanced intermediate (IG) and HG-NHL were treated with the CHOP-M protocol. Mid-cycle (day 10) moderate dose methotrexate (MTX, $300\ mg\ m^{-2}$) and leucovorin rescue were given in combination with a treatment programme derived from CHOP, with intrathecal methotrexate (IT-MTX) as central nervous system prophylaxis (Figure 1). The objective was to determine whether a compromise between the M-BACOD regimen (Skarin *et al.*, 1983) and conventional CHOP could avoid the toxicities of bleomycin and high-dose methotrexate, without compromising efficacy. The results achieved in 110 patients form the basis of this report.

Correspondence: T.A. Lister, ICRF Department of Medical Oncology, St. Bartholomew's Hospital, London EC1A 7BE, UK.
Received 14 December 1992; and in revised form 25 May 1993.

### Patients and methods

### *Patients*

Between June 1980 and July 1986, 118 consecutive, newly diagnosed adults (age $>15$ years) with biopsy proven IG or HG-NHL (confirmed by one of us, AGS) were treated with CHOP-M. There were no patients with Burkitt lymphoma or HTLV-1 associated NHL. Eight patients have been excluded from the analysis; five because of procotol violation and three because of concurrent second malignancy (Hodgkin's disease, two; gastric carcinoma, one).

Clinicopathological characteristics are shown in Tables I and II. Patients with stage IE, II and IIE disease who had 'bulky' disease (a mass $\geqslant 10$ cm in diameter or a mediastinal mass $\geqslant 1/3$ of the intrathoracic diameter), multiple sites of involvement, or gut involvement considered unsuitable for radiotherapy were included. Multiple extranodal sites were involved in 40/71 (56%) patients with Stage IV disease, the liver being the most common site (22 patients). Liver involve-

**Figure 1** Treatment schedule I/T = intrathecal.

**Table I** Clinico-pathological characteristics

| Characteristic | Number of PTS (%) |
|---|---|
| *Gender* | |
| Male | 71 (64) |
| Female | 39 (36) |
| *Age (Years)* | |
| Median (Range) | 54 (15–79) |
| >60 | 38 (36) |
| *Stage* | |
| IE | 8 (7) |
| II | 13 (12) |
| IIE | 17 (15) |
| III | 15 (14) |
| IV | 57 (52) |
| *B Symptoms* | 71 (65) |
| *'Bulk'* | 34 (31) |
| *Sodium* (mmol $l^{-1}$) | |
| 0–136 | 35 (32) |
| >137 | 75 (68) |
| *Albumin* (g $l^{-1}$) | |
| 0–32 | 24 (22) |
| 33–39 | 36 (33) |
| >40 | 50 (45) |
| $\beta_2$ *Microglobulin* (ng $ml^{-1}$) | |
| 0–3 | 49 (18) |
| >3 | 41 (37) |
| Not known | 20 (18) |

**Table II** Histological diagnoses

| Working formulation | | Histology (Kiel) | |
|---|---|---|---|
| F | 1 (1) | Centroblastic | 51 (47) |
| G | 50 (45) | HG-Unclassified | 15 (14) |
| H | 17 (15) | Large cell anaplastic | 7 (6) |
| I | 4 (4) | High grade T Cell | 7 (6) |
| J | 4 (4) | Immunoblastic | 6 (5) |
| Miscellaneous | 7 (6) | Lymphoblastic B | 4 (4) |
| | | Lymphoblastic T | 2 (2) |
| | | Lymphoblastic unclassified | 1 (1) |
| | | Sclerosing mediastinal B cell | 1 (1) |

When these patients were originally treated the diagnosis was made in terms of the Kiel Classification as shown above. The slides were subsequently reviewed by AGS in terms of the Working Formulation. At that time slides for 16 patients were not available, all had originally been classified as high grade lymphoma (10 immunoblastic, 3 centroblastic, 1 lymphoblastic T & 2 high grade unclassified). Both classifications are therefore shown. Histology for 22 patients defied classification according to the Working Formulation. In 6/16 cases in which slides were not available for review, AGS did not feel that Kiel histology could be directly changed to Working Formulation.

ment was deemed to be present if the liver was palpable with two abnormal LFTs, or, if the CT scan showed focal defects. Bone marrow (BM, 11 patients) and gastro-intestinal (GI) tract involvement (13 patients) were pathologically proven in all cases but other sites were not routinely biopsied if there was evidence of disease at a more accessible site. One patient presented with a fifth cranial nerve palsy without lymphoma cells in the cerebro-spinal fluid (CSF).

### *Staging*

The extent of disease at presentation was determined by clinical examination, full blood count, bone marrow aspirate and trephine biopsy, biochemical tests of liver and renal function, and radiography of the chest and abdomen supplemented by computed axial tomography (CT). Patients underwent laparotomy when necessary as a diagnostic procedure or to relieve obstruction, but not solely for staging purposes. Patients were not routinely re-staged during therapy, except when this was considered necessary to determine management. Formal restaging was undertaken one month after completion of therapy by repeating all previously abnormal tests including BM biopsy but other sites were not biopsied to document CR pathologically.

### *Treatment*

The drug doses and schedule for CHOP-M are shown in Figure 1. The first 14 patients received intravenous methotrexate (300 mg $m^{-2}$) on days 8 and 15 of each cycle. This was subsequently changed because of unacceptably severe mucositis. In debilitated, elderly patients (>65 years old), drug doses were reduced by 25–50% from the outset and escalated later according to tolerance. Similar reductions were instituted in a few patients following recurrent, life threatening infections. Intrathecal methotrexate (12.5 mg on Day 1) was given with each cycle of treatment as CNS prophylaxis.

The plan was to administer six cycles of therapy to all patients at 3 weekly intervals. Treatment was delayed by 1 week in the presence of severe neutropenia (absolute neutrophil count $<1 \times 10^9\, l^{-1}$) or severe mucositis and reinstituted with a 50% reduction in the dose of myelosuppressive drugs for that cycle, if a further 1 week delay was necessary. Two responding patients received an extra two cycles because of residual radiological abnormalities at the completion of six cycles of therapy. Two patients received consolidation radiotherapy to the site of bulky mediastinal disease.

'Dose intensity' was calculated for the five drugs for each patient by dividing the total dose given by the time in which treatment was administered. This was then converted to a percentage of the intended 'dose intensity' (six cycles at full dose every 3 weeks). An overall percentage 'dose intensity' was obtained by averaging the five percentage dose intensities for the different drugs. Dose intensities were not calculated for the 22 patients who died before completion of therapy because only 6/22 received more than one cycle of treatment. For seven patients, detailed dosage data were not available, and these patients were thus excluded from the calculation.

### *Definition of response*

(i) Patients in whom 'clinical remission' was achieved were subdivided into two categories:

Complete Remission (CR): patient in normal health with no clinical, biochemical, haematological or radiological abnormalities.

Good Partial Remission (GPR): patient in normal health with no clinical evidence of disease but persistent, equivocal radiological abnormalities (for example, equivocal mediastinal or para-aortic lymph node enlargement), the significance of which is uncertain.

(ii) The response of patients in whom clinical remission was not achieved was regarded as Poor Partial Remission (PPR) provided that the tumour volume was reduced by at least 50%. Any response less than PPR or progression of disease during treatment was documented as treatment failure.

### *Statistical methods*

Survival was calculated from the first day of treatment until death and duration of remission from the date of documented remission to the time of objective evidence of relapse or progression. Overall survival and remission duration curves were plotted according to the method of Kaplan and Meier (1958) and the log rank method (Peto *et al.*, 1977) was used to test for significance of differences in survival distributions. A stepwise regression method based on Cox's proportional hazards model (Cox, 1972) was used to perform multivariate analyses to determine the significance of prognostic factors affecting duration of remission and survival. A stepwise logistic regression model was used to examine the significance of factors affecting achievement of remission. The following 'patient factors' were analysed: age, stage, fever, weight loss, Kiel histology, WF histology, Immunophenotype (B *vs* T), hepatomegaly, bone marrow infiltration, gut involvement, serum albumin, sodium and $\beta 2$ microglobulin, and 'bulk', together with the treatment factors: number of cycles to outcome, and the average dose/cycle of Doxorubicin, Cyclophosphamide and Methotrexate. LDH was not in the past measured routinely at St Bartholomew's Hospital, it has therefore not been evaluated as a prognostic factor, nor has performance status. Hypoalbuminaemia and hyponatraemia have thus been used as surrogates for the latter. Data were analysed using the BMDP and SUREAL statistical software package.

### Results

### *Outcome of therapy (Table III)*

'Clinical remission' was achieved in 70/110 (64%) of patients overall: CR 45 (41%), GPR 25 (23%). Sixty-eight per cent of patients entering clinical remission had no clinical evidence of disease after three cycles of therapy. There was unequivocal persistent disease in 18 patients at completion of therapy (PPR 5, failure 13) although most had shown a degree of response at some stage. None of these patients subsequently had a durable response with alternative regimens and all but one patient (who survived 15 months) died within 1 year.

Three factors, hypoalbuminaemia, hyponatraemia and advanced age correlated with failure to enter clinical remission on multivariate analysis; elderly patients (aged $>50$ years) with either a low serum albumin ($<33\ g\ l^{-1}$) or a low serum sodium ($<137\ mmol\ l^{-1}$) had a clinical remission rate of 17% (5/30) compared with 81% (65/80) in the remaining patients ($P<0.0001$). The presence of B symptoms and liver involvement also had a negative impact ($P=0.02$ and 0.012 respectively). However, both of the latter factors correlated with low albumin and sodium levels and were thus not independent predictors of response. None of the other factors considered were significant.

The mean percentage 'dose intensity' for each of the drugs is shown according to response in Table IV. 'Dose intensities' were the same for patients in whom CR and GPR was achieved and for those in whom treatment failed, though the latter group probably received less Methotrexate ($P=0.02$, *t*-test). However, the five patients in whom treatment resulted in a PPR had significantly lower 'dose intensities' for all the drugs except Vincristine (*P* values shown in Table IV). For patients achieving CR and GPR, the lowest overall 'dose intensity' was 50%, only five patients having values lower than 70%. Patients aged $>60$ had significantly lower mean

**Table IV** Mean % 'dose intensity' by response to therapy

| | *Dox* | *Cyclo* | *Vinc* | *Pred* | *Mtx* | *Total* |
|---|---|---|---|---|---|---|
| CR | 93.1 | 91.0 | 91.6 | 86.1 | 83.2 | 89.0 |
| GPR | 91.9 | 92.6 | 95.0 | 92.4 | 89.5 | 92.3 |
| PPR | 65.2 | 66.5 | 79.9 | 63.6 | 50.2 | 65.1 |
| Prog. Dis. | 82.9 | 84.6 | 88.9 | 89.1 | 62.1 | 80.3 |
| All groups | 89.8 | 89.1 | 91.5 | 86.1 | 80.3 | 87.3 |
| CR + GPR + Prog. Dis. *vs* PPR, *P* = | 0.0006 | 0.003 | 0.12 | 0.009 | 0.02 | 0.001 |

% dose intensities than those aged $<60$ (78.6% *vs* 90.8%, $P<0.01$, *t*-test).

Twenty two patients died before therapy could be completed resulting in a treatment related mortality of 20%. The majority (19/22) were over 50 years of age (median age 63 years) with advanced disease and poor performance status. Most were also found to be hyponatraemic and hypoalbuminaemic. Bulky disease at presentation was present in 12/22 and ten required a laparotomy for diagnosis. One young man with gastro-oesophageal disease had had two laparotomies prior to treatment and died of oesophageal rupture at the site of disease resolution, with no evidence of residual disease at autopsy. Twelve of the 22 patients who died 'early' did so of infective complications whilst pancytopenic. However, at autopsy, 6/22 patients had no evidence of disease; a further six were obviously responding although autopsy was not performed.

### *Duration of remission (Figure 2)*

Forty-seven out of 70 (67%) patients entering clinical remission remain well, 19 have developed recurrent disease, four died in remission. Apart from four late recurrences (at 3½, 4, 5½ and 7 years), all occurred within the first 2½ years. Only three of the patients with recurrent disease are alive, the majority having died within 6 months of recurrence without a durable response to second or third line chemotherapy having been achieved. Four other patients in whom a second complete remission was achieved subsequently received Cyclophosphamide and whole body irradiation with autologous bone marrow transplantation. One died of pneumonia on day 10, two died following further recurrence and one in whom the histology had originally been centroblastic but at the time of recurrence showed follicular lymphoma, remains well at 6 years.

Recurrence always involved at least one of the initial sites of disease. There has been only 1 isolated CNS recurrence to date in the patient (mentioned above) who originally presented with a fifth nerve palsy without cerebrospinal fluid involvement. The recurrence presented rapidly with a radiculopathy, but no evidence of malignant cells in the CSF. The patient eventually died of clinically progressive CNS disease as well as systemic lymphoma. Four patients died in remission; two of cardiac failure, one of pneumococcal septicaemia having had a splenectomy and another who developed acute leukaemia within 2 years of treatment and died without recurrence of lymphoma.

The only factors correlating unfavourably with duration of remission by multivariate analysis were hyponatraemia ($<137$) and advanced aged ($>50$) (Table V). (Hypoalbuminaemia was significant on univariate analysis but correlated with hyponatraemia). There was a trend for longer duration of remission in patients in whom CR as opposed to GPR was achieved, although this did not reach statistical significance. There was no correlation between remission duration and dose intensity for any of the drugs, individually or in combination, on either univariate or multivariate analysis. In contrast, there was a trend on both univariate and multivariate analysis for patients receiving five or more cycles of treatment to have more durable remissions than those receiving four or less, though this did not reach statistical significance ($P=0.08$, univariate; $P=0.05$ multivariate).

**Table III** Outcome of therapy

| | *Number* | *(%)* |
|---|---|---|
| *Clinical remission* | 70 | (64) |
| CR | 45 | (41) |
| GPR | 25 | (23) |
| *Persistent disease* | 18 | (16) |
| PPR | 5 | (5) |
| Fail | 13 | (12) |
| *Death* | 22 | (20) |
| NED[a] | 6 | (5) |
| Lymphoma present[a] | 1 | (1) |
| NA | 15 | (14) |
| Total | 110 | |

[a]As defined at autopsy.
NA Not fully assessable, i.e. autopsy not performed but six patients had evidence of marked clinical response.
NED No evidence of disease.

**Figure 2** Duration of remission for patients in whom 'clinical remission' was achieved.

**Table V** Univariate and multivariate analyses of factors affecting duration of remission and survival

| *Adverse factor* | *Analysis* | *Remission duration* | | *Overall survival* | |
|---|---|---|---|---|---|
| | | *P* | $\chi^2$ | *P* | $\chi^2$ |
| Low | U/V | 0.009 | 6.9 | <0.0001 | 49.9 |
| Albumin[a] | M/V | NS | 5.7 | 0.002 | 9.5 |
| Advanced | U/V | 0.1 | – | <0.0001 | 16.6 |
| Age[b] | M/V | 0.1 | 6.1 | 0.0001 | 22.8 |
| Low[c] | U/V | 0.007 | 7.3 | <0.0001 | 54.8 |
| Sodium | M/V | 0.002 | 9.2 | <0.0001 | 27.7 |
| Advanced[d] | U/V | NS | – | 0.007 | 7.25 |
| Stage | M/V | NS | | NS | |
| Gender | U/V | NS | – | NS | – |
| | M/V | NS | – | 0.002 | 9.9 |
| Kiel Hist | U/V | NS | – | NS | – |
| (lb + cb *vs* rest) | M/V | NS | – | 0.03 | 4.6 |
| β2M | U/V | 0.04 | 4.21 | <0.001 | 10.83 |
| (<3 *vs* >3) | M/V | NS | – | NS | – |
| Bulk | U/V | NS | – | NS | – |
| | M/V | NS | – | NS | – |

Abbreviations: U/V = Univariate; M/V = Multivariate analysis; NS = not significant.[a]Serum albumin ($g\,l^{-1}$): <32 v 33–39 v ⩾40. [b]Age (years): <50 v ⩾50. [c]Serum sodium $mmol\,l^{-1}$: <137 v >137. [d]Stage: I and II *vs* III and IV.

*Survival (Figures 3, 4, 5, 6 and 7)*

Fifty patients (45%) are alive with a minimum follow up of over 4 years and a median follow up of 6¼ years (Figure 3). Four patients died in remission (vide supra). Survival according to stage is shown in Figure 4; on univariate analysis, survival was better for patients with stage I and stage II disease, but this difference was not maintained on multivariate analysis. The median survival of those in whom clinical remission (CR + GPR) could not be achieved was less than 1 year; all died within 2 years, none achieving a durable remission with second line therapy. There was no significant difference in survival beween patients in whom CR as compared to GPR was achieved (Figure 5). Failure free survival is shown in Figure 6.

Apart from response to therapy (i.e. CR + GPR *vs* PPR or no response), by univariate analysis, age, serum albumin, serum sodium, liver involvement and the presence of B symptoms correlated with survival (Table V). However, by multivariate analysis hypoalbuminaemia, hyponatraemia, advanced age, male gender and histology other than diffuse large cell and large cell, immunoblastic (Working Formulation) had an adverse effect on survival as independent variables. The significance of histology ($P = 0.07$) was considerably less than that of the other four variables ($P$ values all $<0.002$).

The four main factors were then used to define three distinct subgroups whose survival differed markedly (Figure 7). Thus, in group A (patients with sodium and albumin values above 136 and 32 respectively), there were no 'early deaths' and the actuarial survival at 5 years was excellent at 85%. In contrast, in group C (either albumin <32 or sodium <136) only 5/41 patients survived beyond 3 years and 17/41 (42%) died 'early' during treatment. The survival of the remaining patients (group B) was intermediate, with an actuarial five year survival of 42%.

*Toxicity*

All patients developed alopecia and the majority experienced nausea or vomiting with the first day of each treatment cycle. Approximately one third of patients developed oral mucositis in relation to the mid-cycle MTX. The majority of patients had a fall in the total white cell count and a concomitant fall in the absolute neutrophil count, the platelet count was relatively spared.

### Discussion

The results presented above, with almost half the initial group of patients alive five years from presentation, and little likelihood of recurrence thereafter, appear better than those previously reported from St Bartholomew's Hospital (SBH) for a broadly comparable group of patients (Gallagher *et al.*, 1982). The disappointment that it has not proved possible to achieve the same excellent results as initially reported with the M-BACOD regimen (Skarin *et al.*, 1983), must however be tempered by the results of the subsequent analysis from Boston, and comparison of the m-BACOD and M-BACOD regimens (Shipp *et al.*, 1990).

Comparison of the overall results for CHOP-M with those for CHOP suggests that it may be as good or marginally

**Figure 3** Overall survival.

**Figure 4** Survival according to stage.

better, but unfortunately, the same may be said when comparison of the 'CHOP' data is made with other major second (Schein *et al.*, 1976; Boyd *et al.*, 1988; Guglielmi *et al.*, 1991) and third generation (Fisher *et al.*, 1987; Gordon *et al.*, 1989; Miller *et al.*, 1990) chemotherapy programmes. Moreover, the recent South West Oncology group randomised study which compared CHOP and the third generation regimens m-BACOD, ProMACE-cytaBOM and MACOP-B showed no advantage in terms of response rate, time to treatment failure or overall survival for the more intensive regimens (Fisher *et al.*, 1993). The findings that 'completeness' of remission did not correlate with survival has important practical implications with regard to decisions made about continuing/stopping therapy when 'equivocal' radiological abnormalities of questionable significance remain.

The most disturbing feature of this study was the treatment-related mortality of 20%, particularly since there was clinical and/or autopsy evidence of response in 12 of these patients. The reported mortality of <10% in two North American (Cabannillas *et al.*, 1983; Klimo & Connors, 1985) and one European series (Guglielmi *et al.*, 1991) contrasts sharply with this experience and with that in a comparable study in which 25% patients died during therapy. Yet, regimens such as M-BACOD, F-MACHOP, ProMACE-MOPP and MACOP-B either contain more drugs or, higher doses of MTX. Thus, the higher mortality observed with CHOP-M and that reported by the Central Lymphoma Group, England (Stuart *et al.*, 1988), may in part have been due to the inclusion of a large proportion of patients with poor prognostic factors. The age distribution in both series was similar.

On multivariate analysis, age was an independent prognostic factor for both achievement of remission and survival. The finding that advanced age (Dixon *et al.*, 1986; Jagganath *et al.*, 1986; Velasquez *et al.*, 1989; Kwak *et al.*, 1990; Hoskins *et al.*, 1991) and hypoalbuminaemia (Coiffier *et al.*, 1991) correlate with a low remission rate has been reported previously, although the relevance of hyponatraemia appears

**Figure 5** Survival of patients in whom CR or GPR was achieved.

**Figure 6** Failure free survival.

new. All of these may reflect general debility and have been used here collectively to represent performance status. A report from the Christie Hospital, Manchester, has shown albumin to be a significant factor affecting survival in high grade NHL (Steward *et al.*, 1984) and more recently, albumin has been shown to influence both overall survival and achievement of CR in intermediate and high grade lymphoma (Cowan *et al.*, 1989). An analysis of serum albumin in Hodgkin's disease showed it to correlate with advanced stage, B symptoms, liver involvement and bulky disease (Gobbi *et al.*, 1985); serum albumin has also been demonstrated to be an important prognostic factor in other malignancies (Kawai, 1973; Osterlind & Anderson, 1986). A multitude of reasons for low albumin levels including reduced synthesis by the liver, and gut loss have been suggested but albumin haemodynamics in malignant lymphoma have not been investigated extensively.

The three major prognostic factors (serum albumin, serum sodium and age) were then used to delineate patient sub groups with markedly different outcomes (Figure 5). Previous studies (Daneiu *et al.*, 1986; Jagganath *et al.*, 1986; Shipp *et al.*, 1986) have used other known prognostic factors to construct similar models but there has been some conflict in the reported importance of individual factors. The recently published International Index defines five features which have been shown to correlate independently with survival, namely: age, stage, performance status, LDH and the number of extra-nodal sites (Shipp *et al.*, 1992). Whether these factors will apply in prospective studies investigating different therapeutic strategies remains to be determined.

Multivariate analysis of factors predicting for freedom from recurrence also revealed advanced age and hyponatraemia at presentation to be significant. Hypo-albuminaemia and elevation of $\beta$-2-microglobulin were significant on univariate analysis but lost significance on multivariate analysis because of very close correlations with the sodium level. It is, of course, not known whether serum sodium would have remained significant had performance status been

**Figure 7** Overall survival according to prognostic groups. Group A = patients with: sodium > 136 mmol $l^{-1}$, albumin > 32 g $l^{-1}$, either males aged < 50 or females Group B = patients not falling into categories A or C Group C = patients with: sodium < 136 mmol $l^{-1}$ or albumin < 32 g $l^{-1}$.

considered since the two are usually closely related. Surprisingly, and in contrast to the findings of others (Shipp *et al.*, 1986; Velasquez *et al.*, 1989; Kwak *et al.*, 1990), the 'bulk' of tumour at presentation was not relevant. Similarly, the amount of chemotherapy received did not influence duration of remission, possibly because so many patients received the majority of treatment as planned.

The precise contribution of Methotrexate to the efficacy of CHOP-M cannot be defined, as the optimal dose in combination chemotherapy is not known, although a clear dose response relationship has been demonstrated in single-agent studies (Tattersall *et al.*, 1975). The lack of any effect of dose intensity suggests that with this regimen, the total amount of therapy administered was probably more important than administration of each cycle strictly on time, or the avoidance of modest dose reduction. However, it should be remembered that 93% of patients received dose intensities > 70% and reductions below this level may still be detrimental.

These results demonstrate the influence of pre-treatment variables on response and survival in a large series of consecutive patients managed at a single centre. Two objective and easily measurable prognostic factors of major importance, serum sodium and serum albumin were identified. It is, however, very clear that for 37 patients in this series this treatment was either inappropriate or inadequate. It is hoped that a flexible approach to treatment incorporating such prognostic indices may result in cure for a greater number of patients without undue toxicity and a better quality of life for those in whom intensive therapy will not be beneficial.

We are pleased to aknowledge the contribution of the medical and nursing staff of Annie Zunz and Dalziel ward, St Bartholomew's Hospital. We thank Dr Amess and the staff of Haematology and Dr Reznek and the staff of Radiology for their help in staging patients. We are grateful to Jane Ashby, Shirley Wragg and Claire Hole for typing many drafts of this manuscript.

### References

ARMITAGE, J.O., DICK, F.R., CORDER, M.P., GARNEAU, S., PLATZ, C. & SLYMEN, D. (1982). Predicting therapeutic outcome in patients with diffuse histiocytic lymphoma treated with cyclophosphamide, adriamycin, vincristine and prednisolone (CHOP). *Cancer*, **50**, 1695-1702.

BERD, D., CORNOG, J., DE CONTI, R., LEVITT, M. & BERTINO, J. (1975). Long term remission in diffuse histiocytic lymphoma treated with combination sequential chemotherapy. *Cancer*, **35**, 1050-1054.

BOYD, D.B., COLEMAN, M., PAPISH, S.W., TOPIBUR, A., KAPEL, S., BERNHARDT, B., FILES, J., SCHWARTZ, S., GAYNOR, M., MCDERMOT, D., REISMAN, A. & COLEMAN, B. (1988). COPB-LAM III: Infusional combination chemotherapy for diffuse large cell lymphoma. *J. Clin. Oncol.*, **6**, 425-433.

CABANNILLAS, F., BURGESS, M.A., BODEY, G.P. & FREIREICH, E. (1983). Sequential chemotherapy and late intensification for malignant lymphomas of aggressive histologic type. *AM. J. Med.*, **74**, 382-388.

COIFFIER, B., BRYON, P.A., FRENCH, M., BLANC, M., SEBBAN, C., BERGER, F. & VIALA, J. (1987). Intensive chemotherapy in aggressive lymphomas: update results of LNH-80 protocol and prognostic factors affecting response and survival. *Blood*, **70**, 1394-1399.

COIFFIER, B., GISSELBRECHT, C., VOSE, J., TILLY, H., HERBRECHT, R., BOSLY, A. & ARMITAGE, J. (1991). Prognostic factors in aggressive malignant lymphomas: description and validation of a prognostic index that could identify patients requiring a more intensive therapy. *J. Clin. Oncol.*, **9**, 211-219.

COLEMAN, M., GERSTEIN, G., TOPILOW, A., LEBOWICZ, J., BERHARDT, B., CHIARIERI, D., SILVER, R.T. & PASMANTIER, M. (1987). Advances in chemotherapy for large cell lymphoma. *Semin. Hematol.*, **24**, 8-20.

COWAN, R.A., JONES, M., HARRIS, M., STEWARD, W., RADFORD, J., WAGSTAFF, J., DEAKIN, D.P. & CROWTHER, D. (1989). Prognostic factors in high and intermediate grade non-Hodgkin's lymphoma. *Br. J. Cancer*, **59**, 276-282.

COX, D.R. (1972). Regression models and life tables. *I.J.R. Stat. Soc. (B)*, **34**, 187-202.

DANIEU, L., WONG, G., KOZINER, B. & CLARKSON, B. (1986). Predictive model for prognosis in advance diffuse histiocytic lymphoma. *Cancer Res.*, **46**, 5372-5379.

DE VITA, V.T., CHABNER, B., HUBBARD, S.M. CANELLOS, G., SCHEIN, P. & YOUNG, R. (1975). Advanced diffuse histocytic lymphoma, a potentially curable disease. *Lancet*, **1**, 248-250.

DE VITA, V.T., HUBBARD, S.M. & LONGO, D.L. (1987). The chemotherapy of lymphomas: looking back, moving forward – The Richard and HIlda Rosenthal Foundation award lecture. *Cancer Res.*, **47**, 5810–5824.

DIXON, D., NEILAN, B. & JONES, S. (1986). Effect of age on therapeutic outcome in advanced diffuse histocytic lymphoma: The Southwest Oncology Group experience. *J. Clin. Oncol.*, **4**, 295–305.

FISHER, R.I., DE VITA, V.T., HUBBARD, S.M., LONGO, D., WESLEY, R., CHABNER, B. & YOUNG, R. (1983). Diffuse aggressive lymphomas: increased survival after alternating flexible sequences of Pro-MACE and MOPP chemotherapy. *Ann. Int. Med.*, **98**, 304–309.

FISHER, R.I., MILLER, T. & DANA, B. (1987). Southwest Oncology Group clinical trials for intermediate- and high-grader non-Hodgkin's lymphomas. *J. Clin. Oncol.*, **24** (2, suppl. 1), 21–25.

FISHER, R.I., GAYNOR, E.R., DAHLBERG, S., OKEN, M., GROGAN, T., MIZE, E., GLICK, J.H., COLTMAN, C. & MILLER, T. (1993). Comparison of a standard regimen (CHOP) with three intensive chemotherapy regimens for advanced non-Hodgkin's Lymphoma. *New Engl. J. Med.*, **328**, 1002–1006.

GALLAGHER, C., COPPLESTONE, A. & MEIKLE, J. (1982). The treatment of disseminated non-Hodgkin's lymphoma of unfavourable histology. *Cancer Chemother. & Pharmacol.*, **8**, 237–241.

GOBBI, P.G., GENDARINI, A., CREMA, A., CAVALLI, C., ATTIERDO-PARRIRELLO, G., FEDERICO, M., DI PRISCO, U. & ASCARI, E. (1985). Serum albumin in Hodgkin's disease. *Cancer*, **55**, 389–393.

GORDON, L., HARRINGTON, D. & GLICK, J. (1989). Randomized phase III comparison of CHOP *vs* m-BACOD in diffuse large cell (DH) and diffuse mixed (DM) lymphoma: equivalent complete response (CR) rates and time to treatment failure (TTF) but greater toxicity with m-BACOD. *ASCO abstracts*, **8**, 255.

GUGLIELMI, C., AMADORI, S., MARTELLI, M., DRAGONI, F. & MANDELLI, M. (1991). The F-MACHOP sequential combination chemotherapy regimen in advanced diffuse aggressive lymphomas: long term results. *Annal. Oncol.*, **2**, 365–371.

HOSKINS, P., NG, V., SPINELLI, J., KLIMO, P. & CONNORS, J. (1991). Prognostic variables in patients with diffuse large-cell lymphoma treated with CHOP-M-B. *J. Clin. Oncol.*, **9**, 220–226.

JAGANNATH, S., VALASQUEZ, W.S. & TUCKER, S.L. (1986). Tumour burden assessment and its implication for a prognostic model in advanced diffuse large cell lymphoma. *J. Clin. Oncol.*, **4**, 859–865.

KAPLAN, E.L. & MEIER, P. (1958). Nonparametric estimation from incomplete observations. *J. Am. Stat. Assoc.*, **53**, 457–481.

KAWAI, T. (1973). *Clinical Aspects of the Plasma Protein*. New York, Springer-Verlag, 222–228.

KLIMO, P. & CONNORS, J.M. (1985). CHOP-M-B chemotherapy for the treatment of diffuse large-cell lymphoma. *Ann. Int. Med.*, **102**, 596–602.

KWAK, L., HALPERN, J., OLSHEN, R. & HORNING, S. (1990). Prognostic significance of actual dose intensity in diffuse large-cell lymphoma: results of a tree-structured survival analysis. *J. Clin. Oncol.*, **8**, 963–977.

MILLER, T., DAHLBERG, S., WEICK, J., FILES, J., EYRE, H., PENDERGRASS, K. & FISHER, R. (1990). Unfavourable histologies of non-Hodgkin's lymphoma treated with ProMACE-CytaBOM: A Southwest Oncology Group study. *J. Clin. Oncol.*, **8**, 1951–1958.

OSTERLIND, K. & ANDERSON, P.K. (1986). Prognostic factors in small cell lung cancer: multivariate model based on 778 patients treated with chemotherapy with or without irradiation. *Cancer Res.*, **46**, 4189–4194.

PETO, R., PIKE, M.C., ARMITAGE, P., BRESLOW, N.E., COX, D.R., HOWARD, S.V., MANTEL, N., MCPHERSON, K., PETO, J. & SMITH, P.G. (1977). Design and analysis of randomised clinical trials requiring prolonged observation of each patient: II analysis and examples. *Br. J. Cancer*, **35**, 1–39.

SCHEIN, P., DEVITA, V., HUBBARD, S., CHABNER, B., CANELLOS, G., BERARD, C. & YOUNG, R. (1976). Bleomycin, adriamycin, cyclophosphamide, vincristine, and prednisolone (BACOP) combination chemotherapy in the treatment of advanced diffuse histiocytic lymphoma. *Ann. Intern. Med.*, **85**, 417–422.

SHIPP, M., HARRINGTON, D., ANDERSON, J., ARMITAGE, J., BONADONNA, G., BRITTINGER, G., CABANILLAS, F., CANELLOS, G., COIFFIER, B., CONNORS, J., DOWAN, R., CROWTHER, D.A & 15 others (1992). Development of a predictive model for aggressive lymphoma: the international NHL prognostic factors project. *N. Engl. J. Med.*, (in press).

SHIPP, M., YEAP, B., HARRINGTON, D., KLATT, M., PINKUS, G., JOCHESLON, M., ROSENTHAL, D., SKARIN, A. & CANELLOS, G. (1990). The m-BACOD combination chemotherapy regimen in large-cell lymphoma: analysis of the completed trial and comparison with the M-BACOD regimen. *J. Clin. Onc.*, **8**, 84–93.

SHIPP, M., HARRINGTON, D.P., KLATT, M., JOCHELSON, M., PINKUS, G., MARSHALL, J., ROSENTHAL, D., DKARIN, A. & CANELLOS, G. (1986). Identification of major prognostic subgroups of patients with large-cell lymphoma treated with m-BACOD or M-BACOD. *Ann. Int. Med.*, **104**, 757–765.

SKARIN, A.T., CANELLOS, G.P., ROSENTHAL, D.S., CASE, D., MACINTYRE, J., PINKUS, G., MOLONEY, W. & FREI, E. (1983). Improved prognosis of diffuse histiocytic and undifferentiated lymphoma by use of high-dose methotrexate alternating with standard agents (M-BACOD). *J. Clin. Oncol.*, **1**, 91–98.

STEWARD, W.P., TODD, I.D.H. & HARRIS, M. (1984). A multivariate analysis of factors affecting survival in patients with high grade histology non-Hodgkin's lymphoma. *Eur. J. Cancer Clin. Oncol.*, **20**, 881–889.

STUART, N.S.A., BLACKLEDGE, G.R.P., CHILD, J.A., FLETCHER, J., PERREN, T.J., O'BRIEN, C.J., JONES, E.L., ELLIS, I.O, KAVANAGH, J.A. & 5 others (1988). A new approach to the treatment of advanced high grade non-Hodgkin's – intensive two-phase chemotherapy. *Cancer Chemother. Pharmacol.*, **22**, 141–146.

TATTERSALL, M.H.N., PARKER, L.M. & PITMAN, S.W. (1975). Clinical pharmacology of high dose methotrexate (NSC-740). *Cancer Chemother. Rep.*, **6**, 25–29.

VELASQUEZ, W., JAGANNATH, S., TUCKER, S., FULLER, L., NORTH, L., REDMAN, J., SWAN, F., HAGEMEISTER, F., MCLAUGHLIN, P. & CABANILLAS, F. (1989). Risk classification as the basis for clinical staging of diffuse large-cell lymphoma derived from 10-year survival data. *Blood*, **74**, 551–557.